# Quantum Entanglement of Monochromatic and Non-Monochromatic Photons on a Waveguide Beam Splitter

**DOI:** 10.3390/e24010049

**Published:** 2021-12-27

**Authors:** Dmitry Makarov, Yuliana Tsykareva

**Affiliations:** Department of Fundamental and Applied Physics, Northern (Arctic) Federal University, nab. Severnoi Dviny 17, 163002 Arkhangelsk, Russia; yu.cykareva@narfu.ru

**Keywords:** quantum entanglement, waveguide beam splitter, von Neumann entropy, monochromatic photons, nonmonochromatic photons, reflection coefficient

## Abstract

It is well known that the waveguide beam splitter can be used as a source for the quantum entanglement of photons. The analysis of such quantum entanglement is a difficult problem even for monochromatic photons, since the system under study is multiparametric. This paper will show that quantum entanglement can be represented in a simple form not only for monochromatic photons but also for non-monochromatic ones. It will be shown that quantum entanglement for non-monochromatic photons can be very different from monochromatic photons, which can be used to create large quantum entanglement.

## 1. Introduction

**Figure 1:**
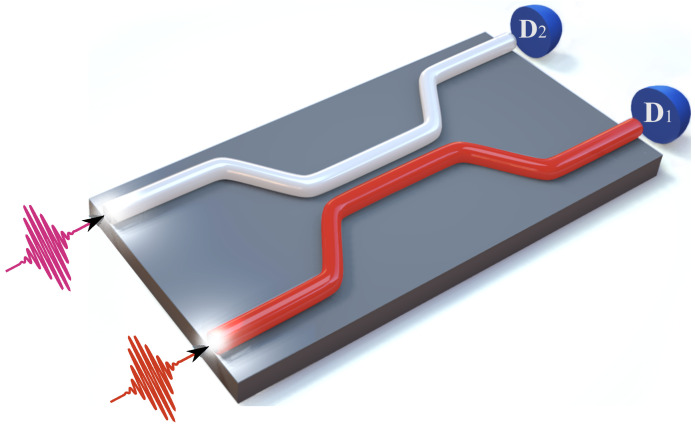
3D representation of the waveguide beam splitter. Photons (in the general case nonmonochromatic) fall on the input ports BS. At the output ports of the BS are detectors $D_{1},D_{2}$ registering photons.

It has long been known that the beam splitter (BS) is a source of quantum entangled photons [1,2,3,4]. Such quantum entanglement sources can be used in many areas of modern quantum technology: quantum metrology [5], quantum information [6], linear optical quantum computing (LOQC) [7,8,9,10], etc. Beam splitters can be of different types. One of the most promising types for quantum technologies is the waveguide BS. Such a BS consists of two connected waveguides, so that the waveguides are brought together close enough to each other that the electromagnetic fields overlap; in this case it is a directional coupler (e.g., [11,12]), see Figure 1. For example, the waveguide beam splitter is an important part in integrated quantum photonics, which is currently implemented in many circuits, e.g., [13,14], and which can be part of an optical quantum computer [9,15].

Theories that describe quantum entanglement of the BS are based on the constancy of its main parameters: reflection coefficient *R* and transmission *T*, where R+T=1, see e.g., [1,3,4,5,16,17,18,19]. Indeed, through these approaches it is possible to show that the BS is a source of quantum entangled photons. Despite this, these approaches are difficult to analyze and interpret, since the resulting expressions are not always simple. Moreover, all these approaches are based on the fact that monochromatic photons are fed to the BS input ports. In these theories, even if we take into account that non-monochromatic photons are fed to the input ports of the beam splitter, the results will be the same as in the case of monochromatic photons. This is due to the fact that in these theories coefficients *R* and *T* are always constant for the waveguide BS. Recently, [20,21] the theory of a frequency-dependent BS in the form of coupled waveguides was presented. In these papers, it was shown that if the BS is represented as a coupled waveguide, the coefficients *R* and *T* depend on the frequencies of the photons fed into both ports of the BS. Taking into account the frequency dependence of the coefficients *R* and *T*, many known theories can be modified, for example, the Hong–Ou–Mandel (HOM) theory of interference [22,23]. It should be added that such a frequency dependence of the coefficients *R* and *T* is inherent only in the [20] waveguide beam splitter. In the case of a prismatic beam splitter, the frequency dependence of the coefficients *R* and *T* does not affect the studied effects [23]. Thus, it is necessary to study the quantum entanglement of photons on the waveguide BS taking into account the frequency dependence of reflection coefficient *R* and transmission *T*. It is also important to present the results in a simple analytical form, without the use of numerical calculations.

In this paper we investigated quantum entanglement of photons on a waveguide BS. For monochromatic photons, the results are presented in a simple analytical form from just one parameter of the system under study, which is the reflection coefficient *R*. In the case of non-monochromatic photons, the results are also presented in an analytical form. It is shown that quantum entanglement in the case of nonmonochromatic photons can be of great importance and is very different from the case of monochromatic photons. Quantum entanglement is analyzed using the von Neumann entropy. Unfortunately, the von Neumann entropy is not always convenient for calculating quantum entanglement because it is hard to calculate.

## 2. Quantum Entanglement of Monochromatic Photons

In the paper [18] it was shown that the wave function of monochromatic photons at the outpu t ports of a waveguide BS can be represented

(1) $$\begin{array}{c} \Psi_{out}=\sum_{k=0}^{s_{1}+s_{2}}c_{k,p}|k,s_{1}+s_{2}-k\rangle , \end{array}$$

where $|k,s_{1}+s_{2}-k\rangle =|k\rangle |p\rangle$ is the state of the photons at the output ports of the BS,

(2) $$\begin{array}{c} c_{k,p}=\sum_{n=0}^{s_{1}+s_{2}}A_{n,s_{1}+s_{2}-n}^{s_{1},s_{2}}A_{n,s_{1}+s_{2}-n}^{*k,p}e^{-2in\;\arccos\left(\sqrt{1-R}\sin\phi \right)},\\ A_{n,m}^{k,p}=\frac{\mu^{k+n}\sqrt{m!n!}}{\sqrt{k!p!}{\left(1+\mu^{2}\right)}^{\frac{n+m}{2}}}P_{n}^{\left(-(1+m+n),m-k\right)}\left(-\frac{2+\mu^{2}}{\mu^{2}}\right),\\ \mu =\sqrt{1+\frac{1-R}{R}\cos^{2}\phi }-\cos\phi \sqrt{\frac{1-R}{R}}, \end{array}$$

where $s_{1}$ is the numbers of photons in the first input port (Fock states) and *k* is the numbers of photons in the first output ports; $s_{2}$ is the numbers of photons in the second input port (Fock states) and *p* is the numbers of photons in the second output ports; $P_{\gamma }^{\alpha ,\beta }\left(x\right)$ is the Jacobi polynomial. Moreover, the number of photons in the system does not change, i.e., the condition $k+p=s_{1}+s_{2}$ [18], where $|k,s_{1}+s_{2}-k\rangle =|k\rangle |p\rangle$ is the state of photons at the output ports of the BS. In this case, the probability $\lambda_{k}\left(R\right)$ of detecting photons in *k* and $p=s_{1}+s_{2}-k$ states at the first and second ports of the BS, respectively, will be $\lambda_{k}\left(R\right)={\left|c_{k,s_{1}+s_{2}-k}\right|}^{2}$. The most important characteristic of a BS is the reflection coefficient *R* and the transmission coefficient *T*, which, as shown in [20,22], will be

(3) $$\begin{array}{c} R=\frac{\sin^{2}\left(\Omega t_{BS}/2\sqrt{1+\varepsilon^{2}}\right)}{\left(1+\varepsilon^{2}\right)};\;T=1-R;\;\cos\phi =-\varepsilon \sqrt{\frac{R}{T}};\;\varepsilon =\frac{\omega_{2}-\omega_{1}}{\Omega }, \end{array}$$

where Ω is a certain frequency characterizing BS and depends mainly on how closely the two waveguides in the beam splitter are connected [20]. As was shown in [22] that $t_{BS}$ is the interaction time of photons in the beam splitter. It has been shown that if the photons are monochromatic and identical then Equation (3) is the same as [11], where $R=\sin^{2}\left(Cz\right)$, ϕ=π/2, C=Ω/(2v) is a constant factor indicating the connection between adjacent waveguides, $z=vt_{BS}$, v is the wave propagation speed in the waveguide); $\omega_{1}$ and $\omega_{2}$—frequencies of incident photons at the first and second ports of the beam splitter, respectively. It should be added that the coupling coefficient in the waveguide *C* is proportional to the frequency Ω, i.e., the greater the coupling in the waveguide, the greater the value of Ω and vice versa. Thus, the coupling in the waveguide is regulated by changing the parameter Ω.

In the given Equations (1)–(3) you can find BS quantum entanglement. Let us choose von Neumann entropy $S_{N}$ as a measure of quantum entanglement. It is well known [20,21,24,25,26] that in this case the entropy will be $S_{N}=-\sum_{k}\lambda_{k}\ln\left(\lambda_{k}\right)$.

Let us represent quantum entanglement $S_{N}(|s_{1}\rangle ,|s_{2}\rangle )$ in a simple form for some initial states $s_{1},s_{2}$:

- for $s_{1}=1$ and $s_{2}=1$
  (4) $$\begin{array}{c} S_{N}(|1\rangle ,|1\rangle )=-{\left(1-2R\right)}^{2}\ln{\left(1-2R\right)}^{2}-4R\left(1-R\right)\ln\left(2R(1-R)\right), \end{array}$$
- for $s_{1}=0$ and $s_{2}=2$ (similarly for $s_{1}=2$ and $s_{2}=0$)
  (5) $$\begin{array}{c} S_{N}(|0\rangle ,|2\rangle )=2\left(-1+R\right)\left(R\ln2+\ln\left(1-R\right)\right)-2R\ln R, \end{array}$$
- for $s_{1}=2$ and $s_{2}=2$
  (6) $$\begin{array}{c} S_{N}(|2\rangle ,|2\rangle )=12{\left(1-2R\right)}^{2}\left(-1+R\right)R\ln\left(-6{\left(1-2R\right)}^{2}\left(-1+R\right)R\right)-\\ 12{\left(-1+R\right)}^{2}R^{2}\ln\left(6{\left(-1+R\right)}^{2}R^{2}\right)-{\left(1+6\left(-1+R\right)R\right)}^{2}\ln\left({\left(1+6\left(-1+R\right)R\right)}^{2}\right), \end{array}$$
- for $s_{1}=1$ and $s_{2}=3$ (similarly for $s_{1}=3$ and $s_{2}=1$)
  (7) $$\begin{array}{c} S_{N}(|1\rangle ,|3\rangle )=6{\left(1-2R\right)}^{2}\left(-1+R\right)R\ln\left(-6{\left(1-2R\right)}^{2}\left(-1+R\right)R\right)+\\ 4{\left(-1+R\right)}^{3}R\ln\left(-4{\left(-1+R\right)}^{3}R\right)+4R^{2}\left(-1+R\right)R\ln\left(-4\left(-1+R\right)R^{3}\right)-\\ R^{2}{\left(3-4R\right)}^{2}\ln\left(R^{2}{\left(-3+4R\right)}^{2}\right)-{\left(1-5R+4R^{2}\right)}^{2}\ln\left({\left(1-5R+4R^{2}\right)}^{2}\right). \end{array}$$

**Figure 2:**
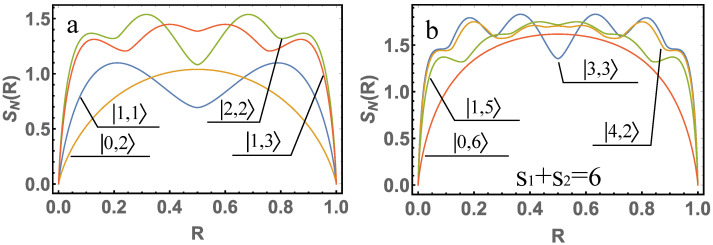
(**a**) The von Neumann entropy is presented for the cases described in Equations (4)–(7). (**b**) The von Neumann entropy is presented for such $s_{1},s_{2}$, when $s_{1}+s_{2}=6$. In the figures, the initial states are selected as $|s_{1},s_{2}\rangle$.

Let us depict in Figure 2 the given functions, as well as the von Neumann entropy for other cases. It is easy enough to find the maximum values of quantum entanglement using the obtained Equations (4)–(7): $[S_{N}(|1\rangle ,|1\rangle {)]}_{max}=\ln3$ with $R=1/2(1\pm 1/\sqrt{3})$; $[S_{N}(|0\rangle ,|2\rangle {)]}_{max}=3/2\ln2$ with R=1/2; $[S_{N}(|2\rangle ,|2\rangle {)]}_{max}=1.5381$ with R=1/2(1±0.31623); $[S_{N}(|1\rangle ,|3\rangle {)]}_{max}=1.4478$ with R=1/2(1±0.1001).

It should be added that here we give only some values for quantum entanglement depending on the initial states $s_{1},s_{2}$. For any other value can be directly calculated using Equation (2) and the von Neumann entropy $S_{N}$. Unfortunately, the von Neumann entropy is not always convenient for calculating quantum entanglement because it is hard to calculate. It is well known that a measure of quantum entanglement can also be the Schmidt parameter K=1/P, where $P=Tr\left(\rho^{2}\right)=\sum_{k}\lambda_{k}^{2}$ which is the purity of the system under study. This measure of quantum entanglement is easier to calculate compared to the von Neumann entropy. For example, in our case it is quite simple to calculate the purity of the system $P(s_{1},s_{2},R)$ for the case of Holland–Burnett (HB) states [18,27], i.e., for $s_{1}=s_{2}=s$ at R=1/2

(8) $$\begin{array}{c} P\left(s,s,1/2\right)=\frac{\Gamma {\left(s+\frac{1}{2}\right)}^{2}{}_{4}F_{3}\left(\frac{1}{2},\frac{1}{2},-s,-s;1,\frac{1}{2}-s,\frac{1}{2}-s;1\right)}{\pi {\left(s!\right)}^{2}}, \end{array}$$

where Γ(x) is the gamma function, ${}_{4}F_{3}\left(x_{1},x_{2},x_{3},x_{4};y_{1},y_{2},y_{3};1\right)$ is the generalized hypergeometric function. It is also not difficult to calculate the purity of the system *P* for the case $s_{2}=0$ for any *R*

(9) $$\begin{array}{c} P\left(s_{1},0,R\right)={\left(1-R\right)}^{2s_{1}}{{}_{2}F}_{1}\left(-s_{1},-s_{1};1;{\left(\frac{R}{1-R}\right)}^{2}\right), \end{array}$$

where ${{}_{2}F}_{1}\left(x,y;z;a\right)$ is Gaussian hypergeometric function. By analyzing the Equation (9) for extremum, we can obtain the maximum of this function at R=1/2. Moreover, at R=1/2 a simple expression for quantum entanglement can be obtained in the form

(10) $$\begin{array}{c} K_{max}=2^{2s_{1}}\frac{{\left(s_{1}!\right)}^{2}}{(2s_{1})!}. \end{array}$$

From Equation (10) you can also find out parameter K for large values of quantum number $s_{1}$, we get $K_{max}\left(s_{1}\gg 1\right)\to \sqrt{\pi s_{1}}$.

## 3. Quantum Entanglement of Non-Monochromatic Photons

It is well known that the wave function $\Psi_{out}$ accounting for non-monochromaticity of photons can be represented as [20,21,28,29]

(11) $$\begin{array}{c} \Psi_{out}=\sum_{k=0}^{s_{1}+s_{2}}\int \phi \left(\omega_{1},\omega_{2}\right)c_{k,p}|k,s_{1}+s_{2}-k\rangle d\omega_{1}d\omega_{2}, \end{array}$$

where $|k,s_{1}+s_{2}-k\rangle =|k\rangle |p\rangle$ is determined similarly to monochromatic photons, i.e., is the state of the photons at the BS output ports; $s_{1}$ and $s_{2}$ are the initial number of photons at 1 and 2 input ports, respectively, $\phi (\omega_{1},\omega_{2})$ the joint spectral amplitude (JSA) of the two-mode wave function ($\int |\phi \left(\omega_{1},\omega_{2}\right){|}^{2}d\omega_{1}d\omega_{2}=1$), $c_{k,p}$ is determined from Equation (2). In this case, the probability $\Lambda_{k}$ of detecting photons in *k* and $p=s_{1}+s_{2}-k$ states at the first and second ports of the BS, respectively, will be

(12) $$\begin{array}{c} \Lambda_{k}=\int {\left|\phi \left(\omega_{1},\omega_{2}\right)\right|}^{2}\lambda_{k}\left(R\right)d\omega_{1}d\omega_{2},\;\;\lambda_{k}\left(R\right)={\left|c_{k,s_{1}+s_{2}-k}\right|}^{2}. \end{array}$$

Let us study the quantum entanglement of the system in question. To see how BS leads to the quantum entanglement of photons we will assume that there is no quantum entanglement at the BS input ports. This is a natural assumption, since we need to study how the BS affects the appearance of quantum entanglement. This means that we will consider the incoming Fock states for photons, but the photons are not monochromatic. In this case, as previously well known, the wave function of the photon is factorizable, i.e., $\Psi_{in}=\int \phi_{1}\left(\omega_{1}\right)|s_{1}\rangle d\omega_{1}\int \phi_{2}\left(\omega_{2}\right)|s_{2}\rangle d\omega_{2}$, where $\phi \left(\omega_{1},\omega_{2}\right)=\phi_{1}\left(\omega_{1}\right)\phi_{2}\left(\omega_{2}\right)$. To calculate quantum entanglement, it is natural to use von Neumann entropy $S_{N}=-\sum_{k}\Lambda_{k}\ln\left(\Lambda_{k}\right)$ [1,20,21,24,25,26] Next, let us choose $\phi_{i}\left(\omega_{i}\right)$ (i=1,2) in the most commonly used form, this is a Gaussian distribution

(13) $$\begin{array}{c} \phi_{i}\left(\omega_{i}\right)=\frac{1}{\sqrt{\sigma_{i}}{\left(2\pi \right)}^{1/4}}e^{-\frac{{\left(\omega_{i}-\omega_{0i}\right)}^{2}}{4\sigma_{i}^{2}}}, \end{array}$$

where $\omega_{0i}$ is the mean frequency (expectation) and $\sigma_{i}^{2}$ is the dispersion. Below, we will use the condition that applies to most photon sources, $\omega_{0i}/\sigma_{i}\gg 1$. At first sight it seems that the condition $\omega_{0i}/\sigma_{i}\gg 1$ is sufficient for photons to be considered monochromatic. This is so, but only for $\Psi_{in}$, and for the wave function at the output ports of the beam splitter $\Psi_{out}$ this is no longer true. This is because the coefficients R,T depend on the photon frequencies $\omega_{1},\omega_{2}$, which eventually leads to the dependence of $\Psi_{out}$ on the dispersion $\sigma_{i}$. Indeed, $\phi_{i}\left(\omega_{i}\right)$ at $\omega_{0i}/\sigma_{i}\gg 1$ behaves like the Dirac delta function, and at $\sigma_{i}\to 0$, it turns into the Dirac delta function. In other words, at $\omega_{0i}/\sigma_{i}\gg 1$ any integral $\int \phi_{i}\left(\omega_{i}\right)f\left(\omega_{i}\right)d\omega_{i}\to f\left(\omega_{0i}\right)$ if function $f(\omega_{i})$ has no dependence similar to $\phi_{i}\left(\omega_{i}\right)$. In this case it is easy to show that $\Psi_{in}$ as well as $\Psi_{out}$ will not be different from monochromatic photons. If $\int \phi_{1}\left(\omega_{1}\right)\phi_{2}\left(\omega_{2}\right)f\left(\omega_{2}-\omega_{1}\right)d\omega_{1}\omega_{2}$ function $f(\omega_{2}-\omega_{1})$ has a dependence similar to $\phi_{1}\left(\omega_{1}\right)$ then $\phi_{2}\left(\omega_{2}\right)$ at $\omega_{0i}/\sigma_{i}\gg 1$ does not behave like the Dirac delta function. Our case considered here just corresponds to the case when in $\Psi_{out}$ the function $c_{k,p}$ includes the reflection coefficient *R*, which has a dependence similar to $\phi_{i}\left(\omega_{i}\right)$ at $\omega_{0i}/\sigma_{i}\gg 1$.

**Figure 3:**
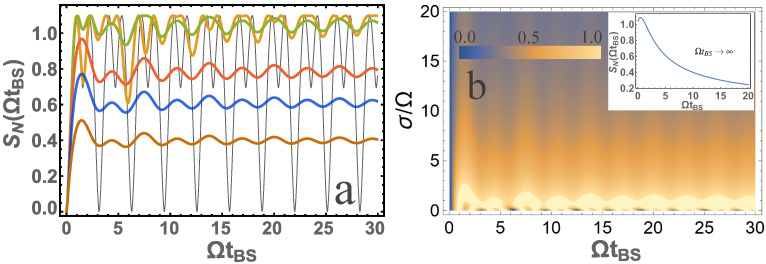
(**a**) The dependence of the von Neumann entropy $S_{N}$ on the parameter $\Omega t_{BS}$ is presented for σ/Ω=10 (brown); σ/Ω=5 (blue); σ/Ω=3 (red); σ/Ω=1 (orange); σ/Ω=1/3 (green); σ/Ω=0 (black). (**b**) A contour plot of von Neumann entropy $S_{N}$ from two system parameters $\Omega t_{BS}$ and σ/Ω is presented. The inset is presented for $S_{N}$ at $\Omega t_{BS}\to \infty$ depending on the parameter σ/Ω. Input photons are in the |1〉,|1〉 state.

Let us show that in the case of non-monochromatic photons, quantum entanglement can be very different from that of the monochromatic photons. Figure 3 represents the dependence of the von Neumann entropy $S_{N}$ according to the dimensionless parameter $\Omega t_{BS}$ for identical photons, i.e., for $\sigma_{1}=\sigma_{2}=\sigma$ and $\omega_{01}=\omega_{02}=\omega_{0}$. Let us compare the results obtained for the monochromatic and non-monochromatic photons.

It should be added that if we use the result for monochromatic and identical photons [11], for the reflection coefficient $R=\sin^{2}\left(\Omega t_{BS}/2\right)$ and, for example, [1] to calculate quantum entanglement, it is easy to obtain the dependencies shown in Figure 3a when σ/Ω=0 (black thin lines). Thus, our result is more general and applicable to non-monochromatic photons. The considered case in Equation (2) is an important case in quantum optics, for example in the case of HOM interference. To analyze this case, it is convenient to represent the contour plot for the von Neumann entropy depending on two parameters σ/Ω and $\Omega t_{BS}$, see Figure 3. From Figure 3b one can see that at sufficiently large $\Omega t_{BS}$ (with $\sigma t_{BS}\gg 1$ also to be satisfied) the quantum entanglement tends to a constant value depending on the σ/Ω parameter. Thus, it is not difficult to find a simple analytical dependence, which is represented by the dependence Figure 3b (inset) as

(14) $$\begin{array}{c} S_{N}=\ln\frac{2{\left(1-J\right)}^{J-1}}{{\left(2J\right)}^{J}},\;J=1+\frac{3}{8}{\left(\frac{\Omega }{\sigma }\right)}^{2}-\frac{\sqrt{\pi }}{16}{\left(\frac{\Omega }{\sigma }\right)}^{3}\left\{3+10{\left(\frac{\sigma }{\Omega }\right)}^{2}\right\}\mathrm{erf}\left(\frac{\Omega }{2\sigma }\right)e^{{\left(\frac{\Omega }{2\sigma }\right)}^{2}}, \end{array}$$

where erf is an error function. From the presented graphs it is clear that the maximum of quantum entanglement will be at σ/Ω=0.445. It is also seen that quantum entanglement is significant and large at σ/Ω∼1, and as σ/Ω increases, it tends to zero.

**Figure 4:**
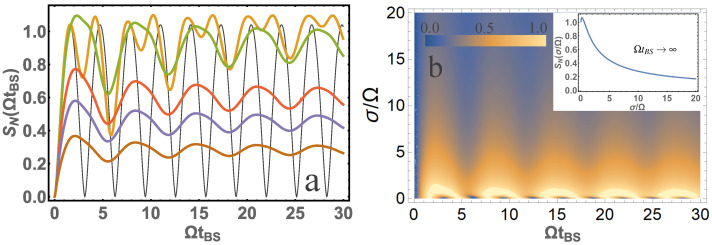
(**a**) The dependence of von Neumann entropy $S_{N}$ on the parameter $\Omega t_{BS}$ is presented for σ/Ω=10 (brown); σ/Ω=5 (blue); σ/Ω=3 (red); σ/Ω=1 (orange); σ/Ω=1/3 (green); σ/Ω=0 (black). (**b**) A contour plot of von Neumann entropy $S_{N}$ from two system parameters $\Omega t_{BS}$ and σ/Ω is presented. The inset is presented for $S_{N}$ at $\Omega t_{BS}\to \infty$ depending on the parameter σ/Ω. Input photons are in the |0〉,|2〉 state.

**Figure 5:**
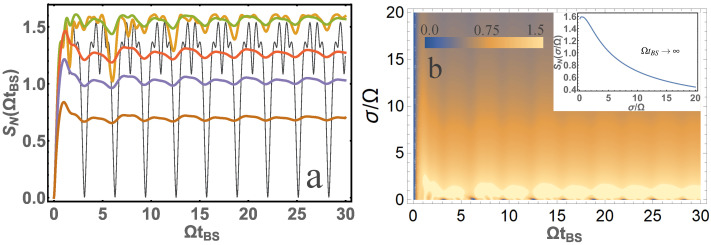
(**a**) The dependence of von Neumann entropy $S_{N}$ on the parameter $\Omega t_{BS}$ is presented for σ/Ω=10 (brown); σ/Ω=5 (blue); σ/Ω=3 (red); σ/Ω=1 (orange); σ/Ω=1/3 (green); σ/Ω=0 (black). (**b**) A contour plot of von Neumann entropy $S_{N}$ from two system parameters $\Omega t_{BS}$ and σ/Ω is presented. The inset is presented for $S_{N}$ at $\Omega t_{BS}\to \infty$ depending on the parameter σ/Ω. Input photons are in the |2〉,|2〉 state.

**Figure 6:**
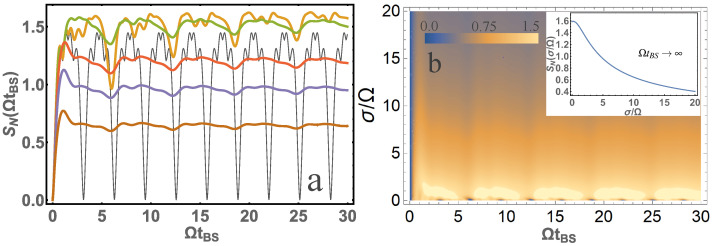
(**a**) The dependence of von Neumann entropy $S_{N}$ on the parameter $\Omega t_{BS}$ is presented for σ/Ω=10 (brown); σ/Ω=5 (blue); σ/Ω=3 (red); σ/Ω=1 (orange); σ/Ω=1/3 (green); σ/Ω=0 (black). (**b**) A contour plot of von Neumann entropy $S_{N}$ from two system parameters $\Omega t_{BS}$ and σ/Ω is presented. The inset is presented for $S_{N}$ at $\Omega t_{BS}\to \infty$ depending on the parameter σ/Ω. Input photons are in the |1〉,|3〉 state.

In Figure 4, Figure 5 and Figure 6 we also present the results of calculations similar to Figure 3, but for other initial states.

Figure 4 shows that the von Neumann entropy at σ/Ω≳1 is quite different from the case of monochromatic photons σ/Ω=0. Figure 4b also shows that the large value of entropy is at σ/Ω∼1. The largest entropy value at $\Omega t_{BS}\to \infty$ would be $S_{N}=1.092$ at σ/Ω=0.24.

Figure 5 shows that the von Neumann entropy also at σ/Ω≳1 is quite different from that of the monochromatic photons. Figure 5b shows that the large value of entropy is at σ/Ω∼1. The largest entropy value at $\Omega t_{BS}\to \infty$ would be $S_{N}=1.591$ at σ/Ω=0.4044.

Figure 6 shows that the von Neumann entropy also at σ/Ω≳1 differs significantly from that of the monochromatic photons. Figure 6b shows that the large value of entropy is at σ/Ω∼1. The largest entropy value at $\Omega t_{BS}\to \infty$ would be $S_{N}=1.595$ at σ/Ω=0.15.

It should be added that one can obtain analytical expressions for the von Neumann entropy at $\Omega t_{BS}\to \infty$, similarly to Equation (14). We do not cite them here, since they are rather cumbersome. One can see in all figures that the value of quantum entanglement is significantly different from the case of the monochromatic photons, i.e., at σ/Ω=0. This is one of the most important conclusions, since previously it has not been taken into account, neither experimentally nor theoretically.

## 4. Discussion and Conclusions

The physical motivation for this work is determined by the fact that it is usually assumed that in beam splitters, regardless of their type, the reflection *R* and transmission *T* coefficients are always considered constant (independent of the photon frequencies) when calculating quantum entanglement. Assuming the coefficients R,T to be constant, quantum entanglement does not depend on whether we consider monochromatic or non-monochromatic photons. Here, we investigated the quantum entanglement of monochromatic and non-monochromatic photons on a waveguide BS. A waveguide BS is frequency-dependent, i.e., reflection coefficients *R* and transmission *T* depend on the frequencies of input photons in ports 1 and 2 of the BS. One of the main conclusions is the essential difference between quantum entanglement of monochromatic and non-monochromatic photons on the waveguide BS. Another important conclusion is that the quantum entanglement is more significant in the case of non-monochromatic photons. If we consider identical incoming photons, it was obtained that the maximum quantum entanglement at σ/Ω∼1. If we choose *R* and *T* as constant values, our theory in the limiting case is σ/Ω→0 (in the general case ε→0 in Equation (3)) and coincides with previously known theories, for example for quantum entanglement [1,18]. Our approach is a more general one that is applicable to for monochromatic (i.e., for constant values of *R* and *T*) and non-monochromatic photons.

Analyzing the results obtained, we can conclude that the waveguide BS can be a good source of quantum entangled photons. Such quantum entanglement can be easily adjusted by changing the Ω parameter, which is the coupling parameter of the two waveguides. This can be done, for example, by separating or bringing the waveguides closer together. It is interesting to note that the waveguide BS can be used as a source for the large quantum entanglement of photons. Such a source generates nearly the maximum possible quantum entanglement at σ/Ω∼1 and $\Omega t_{BS}>1$. Indeed, it is well known that the maximum quantum entanglement for von Neumann entropy $S_{N}=\ln\left(1+N\right)$ when *N* is the total number of photons in a two-part system, e.g., [1,30]. In our case $N=s_{1}+s_{2}$ [1]. In the case of non-monochromatic photons, quantum entanglement is close to its maximum value for identical photons when σ/Ω∼1 and $\Omega t_{BS}>1$. In the case of constants *R* and *T*, quantum entanglement is a periodic function contingent on $\Omega t_{BS}$, and for large $\Omega t_{BS}\gg 1$ a rapidly oscillating dependence, which is a negative factor for use in quantum technologies. It should be added that in [21] the simplest case of |1〉|1〉 input states was analyzed in detail and it was shown that quantum entanglement and photon statistics, at the BS output ports, can be very different for non-monochromatic photons compared with monochromatic photons. In this paper a similar conclusion is drawn, but in the general case, not limited to |1〉|1〉 states. Moreover, in this work simple expressions for quantum entanglement as a function of reflection coefficient *R* were presented for monochromatic photons. Thus, in this paper the main conclusions and results for the calculation of quantum entanglement on the waveguide beam splitter for monochromatic and non-monochromatic photons and their main differences have been presented.

As a result, we can say that the waveguide BS can be used as a source of large quantum entanglement of photons and the obtained results can be used in various fields of quantum technologies.

## Data Availability

Request to corresponding author of this article.
